# Placental Dysfunction Is Associated with Dysregulated Fibrinolytic System Activation

**DOI:** 10.3390/ijms26199339

**Published:** 2025-09-24

**Authors:** Tetiana Yatsenko, Iryna Us, Daria Korolova, Svitlana Zhuk, Halyna Dziuba, Alona Nalbat, Svitlana Kharchenko, Sandor George Vari, Volodymyr Chernyshenko

**Affiliations:** 1 Palladin Institute of Biochemistry of NAS of Ukraine, 01054 Kyiv, Ukraine; 2 Shupyk National Healthcare University of Ukraine, 04112 Kyiv, Ukraine; 3 Kyiv Perinatal Center, 03150 Kyiv, Ukraine; 4 International Research and Innovation in Medicine Program, Cedars–Sinai Medical Center, Los Angeles, CA 90048, USA

**Keywords:** placental dysfunction, hemostasis, fibrinolysis, bleeding, thrombosis

## Abstract

During pregnancy, the maternal hemostatic system undergoes significant changes to support placental angiogenesis, maintain fetal blood flow, and ensure safe delivery. This study investigates the dysregulation of hemostasis in placental insufficiency and explores potential markers for diagnosing and managing this gestational complication. Thromboelastography, coagulation and fibrinolysis functional assays, ELISA, and immunoblotting were employed to assess hemostasis dysregulation in placental dysfunction of two cohorts of pregnant women with placental dysfunction and healthy controls. Thromboelastographic analysis revealed no significant differences in clot lysis indices between the control and placental dysfunction groups, with values remaining within normal ranges, suggesting this method’s limitations for assessing fibrinolysis in pregnancy. The placental dysfunction group demonstrated moderately increased fibrinogen levels and platelet sensitivity to ADP, indicating hemostasis reactiveness. Significantly lower D-dimer levels, decreased plasminogen activator inhibitor activity (total PAI-1 + PAI-2), and increased plasminogen activator activity, driven primarily by uPA in the placental dysfunction group, indicated abnormal fibrinolysis. Immunoblotting confirmed elevated uPA/uPA-PAI complexes and reduced tPA/tPA-PAI complexes, indicating that shutdown of tPA-mediated fibrinolysis and induction of uPA-driven vessel-wall-associated proteolysis are linked to placental dysfunction. Placental dysfunction involves fibrinolytic system dysregulation, marked by decreased PAI and tPA, uPA overproduction, and hypofibrinolysis, contributing to thrombotic risks, impaired placental flow, and complications like fetal growth retardation. PAI/PA ratio and D-dimer levels have diagnostic potential for placental-dysfunction-associated complications.

## 1. Introduction

During gestation, the hemostasis of a pregnant woman undergoes profound changes accompanying placental angiogenesis, maintenance of adequate blood flow to the developing fetus, and delivery [1].

In normal hemostasis, cooperative regulation of coagulation and fibrinolysis prevents bleeding and maintains normal circulation and tissue perfusion. Activation of coagulation results in fibrin polymerization, platelet aggregation, and clot formation. A blood clot induces plasminogen activation on fibrin by tissue-type plasminogen activator (tPA), and newly generated plasmin hydrolyzes fibrin clot and releases fibrin fragments, including D-dimer, into the circulation. Urokinase-type plasminogen activator (uPA) mainly drives fibrinolytic system activation on the cell surface and regulates plasmin activity in cell migration and tissue remodeling. Plasminogen activator inhibitors (PAI-1, PAI-2) regulate clot lifetime, inactivating PA activity (uPA and tPA), and reducing plasmin generation. Disbalance or overactivation of either coagulation or fibrinolytic parts of hemostasis leads to hemorrhagic or thrombotic events, perfusion disorders, and impaired tissue repair process [2].

Normal pregnancy is associated with physiological procoagulant status aimed to minimize blood loss: levels of thrombin, coagulation factors V, X, XI, XII, and antithrombin, protein C, clotting time in activated partial thromboplastin time (aPTT) and prothrombin time (PT) remain largely normal, whereas there is a substantial increase in fibrinogen, D-dimer, and coagulation factors VII, VIII, and IX [3]. The fibrinolytic system of pregnant women also shifts to largely antifibrinolytic regulation with tPA decrease and high activity of PAI-1 and placenta-specific PAI-2 [4]. Fibrinolytic proteins uPA, tPA, and PAI-1,2 are produced by extravillous trophoblast cells, basal and chorionic plates, and chorionic villi tree, where they are extensively involved in vessel net formation, endothelial integrity, placentation process, and placenta detachment during delivery [5].

Alterations in the general and vascular-placental system frequently accompany complicated pregnancy. Pathological changes in the endothelial layer’s structure, integrity, and function during pregnancy are associated with placental insufficiency, preeclampsia, and thrombosis. Impaired vascular-placental remodeling results in a failure of trophic function and fetal hypoxemia, intrauterine growth restriction, prematurity, or fetal demise [6,7].

While many researchers are focused on understanding the role of vascular dysfunction in the pathophysiology of these gestational complications, their mechanisms largely remain unclear. The proteins of the fibrinolytic system not only maintain normal blood flow but also stimulate cytokine release and matrix metalloprotease activation. Therefore, its dysregulation plays a critical role in developing vascular dysfunction [8]. This study aims to establish changes in the fibrinolytic system of pregnant women with placental insufficiency and determine clinically relevant markers of fibrinolysis dysregulation accompanying this gestational complication.

## 2. Results

### 2.1. Characteristics of Patients

In the complicated cohort, placental dysfunction was diagnosed at 12–38 weeks of pregnancy at the admission to the hospital. In total, 9 out of 32 patients had a body mass index (BMI) over 25. Besides placental dysfunction, in the complicated group, there were other adverse characteristics—preeclampsia, gestational hypertension, retrochorionic hematoma, and fetal growth restriction. All the pregnancies in this group were diagnosed with placental blood circulation retardation. Because of hypercoagulation, four complicated patients were prescribed low molecular weight heparins, 2-sulodexide, 2-aspirin, and 1-tranexamic acid.

The control non-complicated cohort had normal placental blood flow and normal coagulation markers.

Overall clinical characteristics of the studied cohorts are presented in Table 1.

### 2.2. Thromboelastographic Analysis

**Figure 1:**
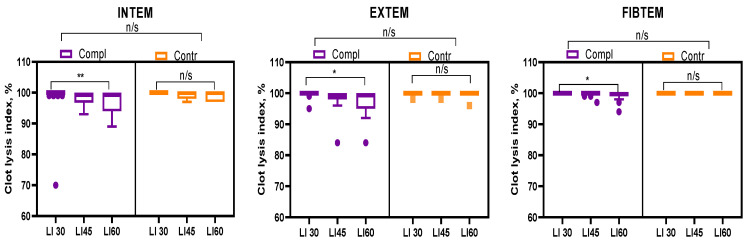
ROTEM clot lysis data of pregnant women with placental deficiency (Compl) compared to the control group with normal pregnancy (Contr). LI30/45/60—clot lysis level at 30/45/60 min after the clotting initiation. INTEM, EXTEM, and FIBTEM methods were used to evaluate intrinsic, tissue factor-induced, and platelet-independent pathways of clot formation. Data is presented as medians with Tukey’s interquartile ranges (IQR). * *p* ≤ 0.05; ** *p* ≤ 0.01; n/s—not significant.

Evaluation of general hemostatic tests, namely thromboelastographic assay, demonstrates no statistically significant difference between control and complicated groups (Figure 1). The clot lysis index of both studied groups was in the normal range (maximum lysis 15%) [9].

Due to the high concentration of fibrinolysis inhibitors, fibrinolysis was almost not observed in thromboelastographic analysis at 30–60 min of blood samples from the control group. Clot lysis in the complicated group was 89–100% INTEM, 84–100% EXTEM, and 94–100% FIBTEM, indicating no hemostatic abnormalities.

The result indicates insufficiency of the original ROTEM test, which is primarily intended to predict not the risks of thrombotic complications but to assess the risks of hemorrhagic syndrome and its targeted prevention and therapy. Thus, it is not suitable for evaluating fibrinolysis in pregnant women with placental disorders.

### 2.3. Coagulation Markers

Activation of the coagulation system triggers fibrinogen conversion to fibrin and platelet activation, resulting in fibrin and platelet clot formation followed by fibrin dissolution by fibrinolytic enzymes. The prothrombin time (International Normalized Ratio—PT INR) and the activated partial thromboplastin time (APTT) characterize extrinsic and intrinsic + common pathways of the clotting cascade, whereas Protein C is a measure of the natural anticoagulant activity in the circulation [3].

The analysis of blood plasma samples revealed that both pregnant women with placental dysfunction and uncomplicated pregnant controls predominantly exhibited normal coagulation markers [3]. The complicated group had higher fibrinogen levels than uncomplicated (Table 2), although, in both cases, the levels remained within the normal range of approximately 300–650 mg/dL. APTT, PT INR, and Protein C levels were in the normal range and did not significantly differ between groups (Table 2).

The platelet component of hemostasis is characterized by the number of platelets and their ability to aggregate in the presence of agonists like adenosine-5-diphosphate (ADP), epinephrine, thrombin, or collagen [10]. We evaluated the platelet component by assessing platelet count, the rate of ADP-induced platelet aggregation, and the lag phase of collagen-induced aggregation (Table 2). The platelet component in both studied groups was within the normal range; platelet count and aggregation in response to the strong agonist collagen did not differ between women with placental dysfunction and the uncomplicated control group. However, ADP-induced platelet aggregation was increased in the complicated group. ADP, a weak primary agonist of platelet activation, may indicate a reactive platelet state in response to placental insufficiency and impaired endothelial function [10].

**Figure 2:**
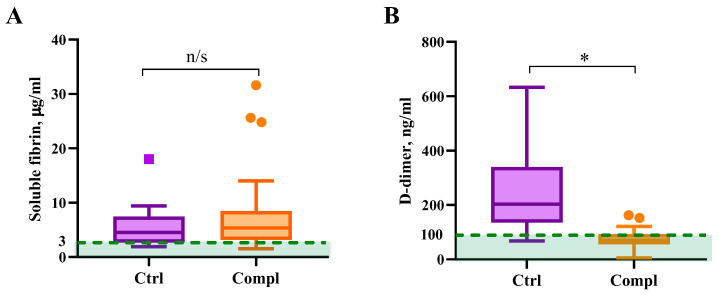
Soluble fibrin (**A**) and D-dimer (**B**) concentrations in the blood plasma of pregnant women with placental dysfunction (*n* = 30) compared to the control group with normal pregnancy (*n* = 12). The green area represents the normal range of soluble fibrin (up to 3 μg/mL [12]) and D-dimer (up to 100 ng/mL [13]) in healthy non-pregnant subjects. Data is presented as medians with Tukey’s interquartile ranges (IQR). * *p* < 0.05; n/s—not significant.

Coagulation is accompanied by circulating soluble forms of fibrin in the bloodstream. Fibrinolysis releases fibrin degradation products, among which D-dimer is widely used as a measure of blood clot formation and breakdown [11]. Subsequently, circulating D-dimer alongside soluble fibrin indicates both coagulation and fibrinolysis. Our results demonstrate an increase in soluble fibrin in the circulation of pregnant women with and without complications, compared to the reference values and significantly lower D-dimer in the complicated group (Figure 2).

The normal parameters of soluble fibrin content in the blood samples of healthy non-pregnant adults is below 3 μg/mL [12]. Both non-complicated and complicated studied groups had increased soluble fibrin compared to the reference values for non-pregnant adults, and there was no significant difference between the studied groups (Figure 2A). However, the concentration of soluble fibrin was elevated in comparison to the control group, which indicates the increased procoagulant potential in both groups. Reflecting the formation of active thrombin in blood plasma, soluble fibrin is assumed to be a specific and reliable indicator for diagnosing thrombophilia [14,15].

In the healthy control group, we found that the average level of D-dimer (68–633 ng/mL) was above the normal level of D-dimer in healthy non-pregnant subjects of 100 ng/mL [13], whereas in the group with placental dysfunction, it was significantly lower (5–162 ng/mL) (Figure 2B). Clinical recommendations do not clearly define reference ranges of D-dimer for pregnant women. Chan et al. study indicated a D-dimer range of 210–780 ng/mL for normal pregnancy [16]. Another study demonstrated that more than 25% of pregnant women in the second trimester and almost all the women by the end of the third trimester without any complications have D-dimer levels at or above 500 ng/mL [3]. A review by Abbassi-Ghanavati et al. recommends a reference interval of 130–1700 ng/mL for a normal pregnancy [17].

It is important to emphasize that D-dimer itself does not provide information about the degree of activation of the blood coagulation system. It is rather an indicator of the post-thrombotic state, confirms the existence of fibrin deposits, and indicates the activity of their fibrinolysis [18,19]. Taking this into consideration, we must assume that the decreased D-dimer levels in the complicated group are likely indicative of abnormal changes in fibrinolysis.

### 2.4. Fibrinolytic System Markers

**Figure 3:**
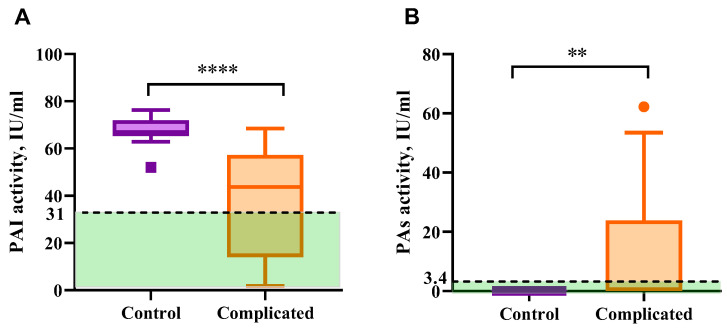
Fibrinolytic system dysregulation markers in the circulation of pregnant women with placental insufficiency. (**A**) The total activity of plasminogen activator inhibitors 1 + 2 (PAI-1 and PAI-2) in blood plasma samples in the complicated pregnancy group (*n* = 30) compared to the control group (*n* = 12). The green area represents the normal range of PAI-1 activity in healthy non-pregnant subjects (0.5–31 IU/mL) [21]. (**B**) The total activity of plasminogen activators (uPA and tPA) in the blood plasma of pregnant women with placental disorders (*n* = 30) and the control group (*n* = 12). The green area represents the normal range of tPA activity in healthy non-pregnant subjects (0–3.4 IU/mL) [22]. Data is presented as medians with Tukey’s interquartile ranges (IQR). ** *p* < 0.01; **** *p <* 0.0001.

Total fibrinolysis inhibitory activity (total activity of PAI-1 + PAI-2) in the blood plasma of pregnant women with placental insufficiency was significantly lower than in the control group with the normal course of pregnancy (Figure 3A). In the group of women with complications of pregnancy, PAI activity was within the range of 1–69 units/mL, while in pregnant women without complications, it was 65–72 units/mL. This decrease is probably due to a lower level of PAI-2, which indicates the deterioration of placental blood circulation [20].

Reduced activity of PAI in the circulation of pregnant women with placental dysfunction is accompanied by the increased total activity of plasminogen activators (PA) (Figure 3B). Normally, identification of plasminogen activators activity requires isolation of euglobulin fraction from blood plasma to avoid PA/plasmin inhibition by other plasma components, thus PA is not identified in whole plasma by plasmin activity measurement. The plasma PA activity in healthy subjects is rather undetectable [23]. In the blood plasma of 43% of pregnant women (13/30) with placental disorders, PA activity was not compensated by the antifibrinolytic system, indicating a possible risk of plasminogen hyperactivation. At the same time, thromboelastographic data do not show hyperfibrinolysis. The predominance of the coagulation process in the whole blood probably reflects the role of cellular components of blood and factors released by blood cells in response to clotting in the hemostatic balance of pregnant women.

**Figure 4:**
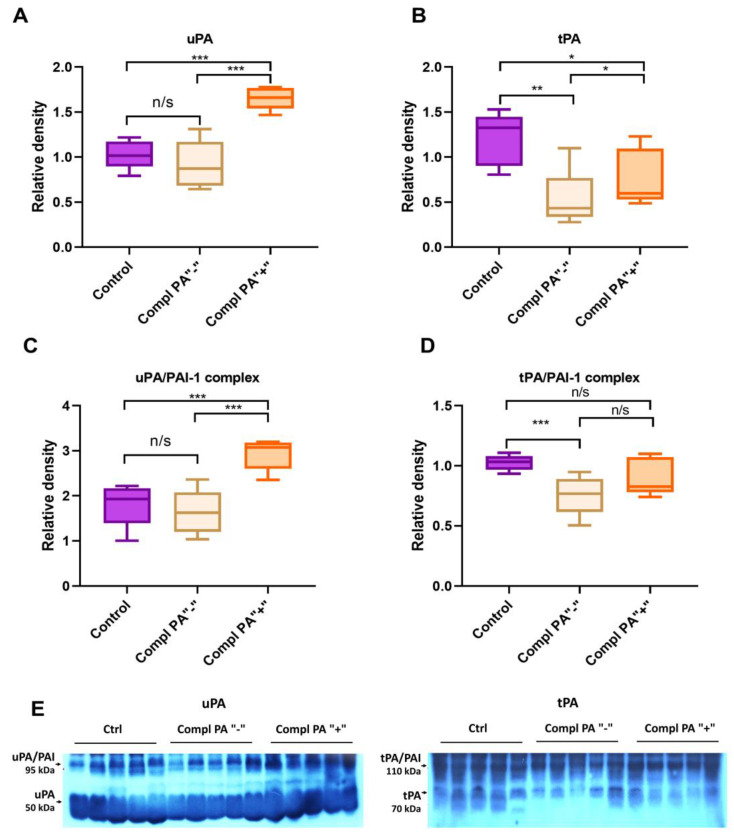
Plasminogen activators and activators/inhibitors complexes level in the blood plasma of pregnant women with normal pregnancy (Control), placental dysfunction with normal PA activity (Complicated PA “-”) and with increased PA activity (Complicated PA “+”). Each group *n* = 5. uPA (**A**) and its complex with PAI-1 (**C**), tPA (**B**), and its complex with PAI-1 (**D**) were measured by Western blot analysis using specific antibodies for human uPA and tPA (**E**). Samples with equal protein amounts were loaded on the gel. The quantification of band intensity normalized to a sample from a healthy donor. Data are presented as medians with Tukey’s interquartile ranges (IQR). * *p* < 0.05; ** *p* < 0.01; *** *p* < 0.001; n/s—not significant.

To understand the nature of the increase in plasminogen activator activity, we compared the levels of uPA, tPA, uPA/PAI, and tPA/PAI complexes in randomly selected plasma samples (*n* = 5 per subgroup) from pregnant women with placental dysfunction, including those with PA activity and those without, and from the control group (*n* = 5) (Figure 4).

Immunoblotting of blood plasma samples demonstrates uPA and uPA/PAI complex levels in the control group and placental dysfunction with normal PA activity was the same, whereas in the complicated pregnancy group with increased PA activity uPA and uPA/PAI levels were 1.5 times higher than in both control and PA “-“ group (Figure 4A,C). A significant reduction in tPA and tPA/PAI complex levels was observed in the blood plasma of pregnant women with placental dysfunction compared to normal pregnancy (Figure 4B,D). In the complicated subgroups, tPA is higher in the group with increased PA activity. According to this data, the development of placental dysfunction is manifested in plasminogen activation system disbalance. The increase in PA activity originates mainly from uPA.

## 3. Discussion

Insufficient effectiveness of preventive therapy of vascular-placental disorders is mostly associated with an insufficient understanding of the mechanisms of development of coagulopathy and the lack of a single point of view on the nature of coagulation disorders and, in particular, local hemostasis. Due to the lack of diagnosis of fibrinolysis in the development of vascular-placental disorders, the prevalence of these disorders is probably higher than described so far. Accurate diagnosis of hemostasis disorders in pregnant women requires a sensitive and proven fibrinolysis test.

Available coagulation tests, in general, are not indicative of hypofibrinolytic disorder. As we demonstrated in this study, TEG lysis indexes do not represent changes in clotting/fibrinolysis balance in the circulation of pregnant women with placental dysfunction. A moderate increase in fibrinogen level and platelet sensitivity to ADP is indicative of placental-dysfunction-induced hemostasis reactiveness.

One of the most promising markers is the simultaneous determination of soluble fibrin, as a known marker of thrombophilia, and the D-dimer as a marker of post-thrombosis and fibrinolytic activity [24]. In our previous study [25], we found that soluble fibrin concentration in blood plasma grows up to 30 µg/mL during normal healthy pregnancy. In normal healthy pregnancy D-dimer gradually increases from 10 to 100 ng/mL in the first trimester to 50–500 ng/mL in the third trimester [25]. This clearly indicates that a healthy pregnancy has a procoagulant status balanced with increased fibrin breakdown. Here, we demonstrated that placental insufficiency is associated with a disbalance of normal procoagulant status and decreased D-dimer, indicating hypofibrinolysis and thrombosis risks.

The increase in plasminogen activator activity in the circulation of pregnant women is possibly linked to placenta abruption [26]. Our data suggests that a change in PAI/PA activities could be indicative of pregnancy complications, and the PAI/PA ratio is a prospective marker of vascular-placental disorders.

PAI-1 is known to be one of the regulators of maternal uterine spiral artery remodeling [27]. Hypoinvasion and disturbed placental vessel remodeling are associated with reproductive system diseases like recurrent pregnancy loss, preeclampsia, fetal intrauterine growth retardation, and even antenatal fetus death [28,29]. Likewise, in some studies, decrease in PAI-2 protein production and activity is associated with placental insufficiency and fetal growth restriction [30,31], while PAI-1 increase is linked to thrombotic risks [32]. Approximately 60% of the PA-inhibitory activity in the plasma during pregnancy is provided by PAI-1 [33]. Lower total PAI activity in complicated pregnancy patients compared to the healthy control group is a sign of placental dysfunction and has diagnostic value as a marker of fibrinolytic imbalance during pregnancy.

The placenta is considered to be the main source of urokinase in a woman’s body during pregnancy, while changes in the level of tissue activator are provided by the mother’s circulatory system [34,35]. The activity of both of the activators in the bloodstream is higher in the first trimester of pregnancy, then drops down during the second and third trimesters and dramatically increases before the labor [36]. The main plasminogen activators, uPA and tPA, have different specificities: uPA generates plasmin mainly on the cell surface and thus is more involved in cell migration and tissue remodeling, while tPA activates plasminogen predominantly on the fibrin surface, providing fibrin deposition lysis initiation [37]. Roes et al. reported an increase in circulatory tPA in the blood of pregnant women with preeclampsia as a marker of endothelial dysfunction [30]. In our study, we found a 50–60% decrease in the blood plasma tPA in the placental dysfunction group, indicating a decrease in fibrin-associated plasminogen activation potential and a possible risk of thrombosis. At the same time, elevated uPA in pregnant women with high total plasminogen activation activity suggests changes in the production of urokinase by placental tissues, leading to increased cell-associated plasmin generation. Significant (100–200 times) increase in uPA production by placental tissues with subsequent increase in uPA activity in blood and amniotic liquid accompanies labor, generating plasmin in placental tissues and triggering its detachment [38]. Increased uPA in the bloodstream of women with placental dysfunction possibly indicates a high risk of preterm placenta detachment and miscarriage.

The fibrinolytic system acquires multidirectional systemic and parietal blood circulation changes during pregnancy. Intravascular fibrinolysis is activated due to a significant increase in urokinase activator of plasminogen and a decrease in alpha-2-antiplasmin, which are responsible for systemic fibrinolysis. Thus, the woman’s body is protected from intravascular thrombus formation before labor. This also explains the phenomenon of an increase in the D-dimer level under conditions of physiological pregnancy, in which, despite shocking procoagulant changes in the hemostasis system, venous thromboembolic complications do not occur. However, due to a significant increase in PAI-1 in comparison with tissue plasminogen activator, which is synthesized in the endothelium, on the contrary, not activation, but inhibition of blood vessel fibrinolysis is ensured, and it usually prevents massive blood loss during childbirth [3,30]. Dysregulation of plasminogen activators and activator inhibitors, namely a substantial decrease in PAI and tPA along with uPA overproduction, is linked to placental dysfunction, deteriorated blood circulation between placenta and fetus, fetal growth retardation, and high risk of miscarriage. Assessment of PAI/PA activity, as well as the balance between soluble fibrin and D-dimer, is a promising instrument for the evaluation of hemostasis disorders and prognosis of pregnancy complications.

## 4. Materials and Methods

### 4.1. Study Design and Patient Groups

The study cohort included 32 pregnant women with placental dysfunction admitted to the Kyiv Perinatal Center (Kyiv, Ukraine). The study period spanned March 2020 to February 2021. All study participants gave informed consent for anonymizing their clinical data.

The inclusion criteria consisted of women aged 18–40 years, either inpatients or outpatients, with a singleton pregnancy complicated by placental dysfunction developing during the second or third trimester. We defined the placental dysfunction by intrauterine growth restriction and impairment of umbilical artery circulation confirmed by ultrasonic fetometry. Fetal growth restriction was defined as uterine growth parameters below the 10th percentile for gestational age according to the Hadlock formula. Placental dysfunction by Dopplerometry was determined from pathological percentile values of blood flow velocity in the umbilical, middle cerebral, and uterine arteries. Impaired circulation of the umbilical artery was defined as zero or reverse blood flow in the artery.

Exclusion criteria: use of antithrombotic therapy during the pregnancy, fetus or newborn death not linked to the placental dysfunction, severe extragenital pathology, chromosomal pathology, or fetal malformations.

Women were admitted to the hospital with the availability of basic medical information, including patient history, initial blood laboratory data, and outcome data.

Twelve healthy women aged 18–45 years with physiologically normal singleton intrauterine pregnancy (II-III trimester of gestation) without thrombotic or hemorrhagic complications were also recruited and served as the control cohort.

All the recruited participants have provided informed consent for the use of their clinical data and blood samples in the present study.

### 4.2. Blood Sample Collection and Plasma Preparation

Blood samples were collected at the time of admission.

Whole citrated blood obtained from venipuncture was used for thromboelastographic analysis.

Blood plasma samples for coagulation and fibrinolysis markers measurement were prepared after whole blood collection into 3.8% sodium citrate tubes and centrifugation at 400× *g* for 10 min at RT without brake. The undiluted plasma was then aliquoted and stored in polypropylene tubes at −80 °C for subsequent analysis.

### 4.3. Methods

#### 4.3.1. Thromboelastography

Thromboelastographic analysis was performed on a ROTEM Delta device using the TEG reagent kit (Instrumentation Laboratory Werfen, Munich, Germany). Citrated whole blood was used for the study. Tests were conducted within two hours of receiving the material.

In the EXTEM test, clotting was initiated by adding tissue thromboplastin (tissue factor). In the INTEM test, blood coagulation was activated by the contact route, as in the APTT tests. This test is sensitive to the deficiency of intrinsic pathway factors.

FIBTEM test was used to analyze fibrin polymerization during clot formation. In this test, the agglutination process was initiated the same way as in the EXTEM test, but the reagent contained cytochalasin D to block platelet aggregation.

The following coefficients were used to evaluate fibrinolysis: LI30, LI45, and LI60 (lysis index after 30, 45, and 60 min, given in %)—represent the percentage of remaining clot stability out of the maximal clot firmness value at 30/45/60 min after clotting time.

#### 4.3.2. Fibrinogen Assay

Fibrinogen concentration in the blood plasma was determined by the modified spectrophotometric method. Blood plasma (0.2 mL) and phosphate-buffered saline (1.7 mL) were mixed in a glass tube. Coagulation was initiated by the addition of 0.1 mL thrombin-like enzyme from *Agkistrodon halys halys* snake venom (1 NIH/mL) to prevent fibrin cross-linking. The mixture was incubated for 30 min at 37 °C. The fibrin clot was removed and resolved in 5 mL of 1.5% acetic acid. The concentration of protein was measured using an OPTIZEN POP spectrophotometer (Daejeon, Republic of Korea) at 280 nm (ε = 1.5) [39].

#### 4.3.3. Protein C Assay

Total PC level in blood plasma was determined using protein C activator and specific chromogenic substrate S2236-p-Glu-Pro-Arg-pNa (Sigma-Aldrich, St. Louis, MO, USA) [40]. In a well of a 96-well plate, 0.02 mL of blood plasma sample, 0.03 mL of S2236 solution (0.25 mM), and 0.03 mL of protein C activator (Siemens, Munich, Germany) solution were mixed in the tris-buffered saline with 0.001 M CaCl_2_ at a final volume of 0.25 mL. The generation of colored product p-nitroaniline was monitored at 405 nm with 492 nm as reference using Thermo Multiskan (Thermo Fisher Scientific, Waltham, MA, USA). Results were presented as percentages from control values.

#### 4.3.4. Activated Partial Thromboplastin Time Assay

Activated partial thromboplastin time (APTT) reagent and control donor blood plasma were from Siemens Healthineers Dade Actin™ Activated Cephaloplastin Reagent set (Munich, Germany). APTT assay was performed using the following procedure: 0.1 mL of studied blood plasma was mixed with an equal volume of APTT-reagent and incubated for 3 min at 37 °C. Then, the coagulation was initiated by adding 0.1 mL of 0.025 M solution of CaCl_2_, and the clotting time was monitored. The clotting time was evaluated using a coagulometer CT2410 (Solar-STS, Kharkiv, Ukraine) [39].

#### 4.3.5. Prothrombin Time Assay

Prothrombin time was measured as follows: clotting was initiated by mixing 0.1 mL of blood plasma with 0.1 mL of 0.025 M CaCl2 and 0.1 mL of thromboplastin reagent, the time of clotting was monitored. Thromboplastin acts through the tissue factor pathway of coagulation and activates only carboxylated and uncleaved forms of prothrombin. The clotting time was evaluated using a coagulometer CT2410 (Solar-STS, Kharkiv, Ukraine). Results were presented as International Normalized Ratio (INR) [39].

#### 4.3.6. Platelet Aggregation Measurement and Platelet Count

Platelet aggregation was measured based on changes in the turbidity of human platelet-rich plasma (PRP). In a typical experiment, 250 μL of PRP was incubated with 0.001 M CaCl_2_ and 12.5 μM adenosine diphosphate (ADP) for ADP-induced platelet aggregation or 10 mg/mL collagen for collagen-induced platelet aggregation at 37 °C. Aggregation was monitored for 10 min using the SOLAR analyzer of platelet aggregation AP2110 model (Solar-STS, Kharkiv, Ukraine). The platelet count was estimated using the same device [39].

#### 4.3.7. Soluble Fibrin Assay

Soluble fibrin (SF) was detected using sandwich ELISA, as described in [12]. Fibrin-specific monoclonal antibody I-3C [41] was used as a catch antibody. Biotinylated monoclonal antibody II-4d with epitope in NH_2_-terminal fragment of γ-chain of D-region of fibrin(ogen) molecule was used as a tag-antibody. As a substrate, 0.1 mg/mL 3,3′,5,5′-Tetramethylbenzidine Dihydrochloride was used in 0.05 M phosphate-citrate buffer pH 5.0 with 0.002 mL 30% H_2_O_2_. The reaction was terminated by adding 0.1 mL of 2 M H_2_SO_4_. The optical density of the solutions was determined at 450 nm with 630 nm as reference using plate reader RT 2100C (Rayto, Shenzhen, China). For the calibration curve for quantitative analysis we used samples of fibrin desA [42].

#### 4.3.8. D-Dimer Measurement

D-dimer was detected using sandwich ELISA as described above for soluble fibrin with the following exception: biotinylated D-dimer-specific monoclonal antibody III-3B that has epitope in NH_2_-terminal fragment of Bβ-chain of D-region of fibrin(ogen) molecule was used as the catch antibody. The concentration of D-dimer was measured using the calibration curve obtained for purified D-dimer [13].

#### 4.3.9. PAI and PA Activity Assay

Plasma total PAI (PAI-1 + PAI-2) levels and plasma total PA (uPA + tPA) were measured using a modified colorimetric method of tPA analysis [43].

The recombinant tissue plasminogen activator (Actilyse, Boehringer Ingelheim, Biberach, Germany) was used to analyze plasminogen activator inhibitor activity and plasminogen activator activity, as well as for polyclonal antibody production.

The activity of PAIs in plasma samples was determined by the decrease in the activity of recombinant tPA, as measured by plasmin generation. Human plasminogen was isolated from healthy donor citrate blood plasma by a standard method [44]. The reaction mixture (100 μL) contained plasma-5 μL (diluted 1:1 with 0.05 M tris-buffered saline pH 7.4 (TBS)), fibrinogen fragment D-120 μg/mL, Glu-plasminogen-40 μg/mL; tPA-5 μL of a solution of 50 IU/mL and chromogenic substrate S_2251_ (final concentration of 0.3 mM). PA activity was measured in blood plasma, diluted five times in TBS pH 7.4 using the same method as for PAI, without exogenous tPA addition. The reaction was carried out at 37 °C in TBS pH 7.4. The absorbance was measured at a wavelength of 405 nm on a Multiscan Titertek plate reader (Labsystems, Helsinki, Finland) after 80 min of the reaction. For the PAI assay, exogenic recombinant tPA was used as standard, and PAI activity (international units per mL, IU/mL) in plasma samples was calculated by a decrease in tPA activity: 1 IU tPA = 1 IU PAI [45]. 

#### 4.3.10. Western Blot Analysis

Plasma levels of tPA, uPA, and the activator complexes with PAI-1 were measured by Western blot analysis.

Rabbit polyclonal antibodies to tissue activator and urokinase were affinity purified by a standard method [46] using Protein A-Sepharose (#17-0780-01, GE Healthcare Life Sciences, Chicago, IL, USA). Recombinant human tissue activator (Actilyse, Boehringer Ingelheim, Biberach, Germany) and human urokinase (Urokinase, Medac, Wedel, Germany) were used as an antigen for animal immunization.

Blood plasma was mixed with 2× Laemmli Sample Buffer and boiled for 1 min. Serum samples (20 or 70 µg proteins) were applied on 10% acrylamide gel, transferred to nitrocellulose membrane (#1215483, GVC, Bologna, Italy), blocked with 5% skim milk for 1 h, and incubated overnight at 4 °C with primary antibody. Membranes were then washed with PBS-T and incubated with a secondary goat antirabbit antibody conjugated with horseradish peroxidase (HRP) (#A16104, Invitrogen, Carlsbad, CA, USA). The detection of protein bands was performed with ECL reagent exposing the membrane to X-ray film.

Quantification of the protein bands was performed with ImageJ bundled with 64-bit Java 8 software (National Institutes of Health, Bethesda, MD, USA). Available online: https://imagej.net/ (accessed on 13 August 2022).

#### 4.3.11. Statistical Analysis

The data analysis was performed with GraphPad Prizm 8 software (GraphPad Software, LLC, San Diego, CA, USA) using the Mann–Whitney test (for non-normally distributed data) and t-test (for normally distributed data) for two group comparison, and one-way ANOVA for three group comparison.

## 5. Conclusions

Placental dysfunction is associated with significant dysregulation of the fibrinolytic system, including a decrease in PAI and tPA, along with uPA overproduction. This imbalance disrupts normal hemostasis, leading to hypofibrinolysis, increased thrombotic risks, impaired placental blood flow, and complications such as fetal growth retardation and miscarriage. Markers like PAI/PA activity ratios, as well as the balance between soluble fibrin and D-dimer, are promising diagnostic tools for assessing these imbalances and predicting pregnancy complications. A better understanding of these mechanisms and improved diagnostic tests are essential for enhancing the prevention and management of vascular-placental disorders.

## Figures and Tables

**Table 1:** Clinical characteristics of pregnant women and the pregnancy course.

	Control (*n =* 12)	Complicated (*n =* 32)
**Maternal Characteristics**		
Age at delivery (years), mean ± SD (range)	31.3 ± 2.7 (29–35)	32.0 ± 6.3 (18–45)
BMI	20.4 ± 2.3 (17–23)	23.6 ± 4.0 (18–31)
Chronic hypertension, *n* (%)	0	1/32 (3.13%)
Tobacco smoking, *n* (%)	0	1/32 (3.13%)
History of preeclampsia, *n* (%)	0	2/32 (6.26%)
Nulliparous, *n* (%)	1/12 (8.3%)	12/32 (37.5%)
**Pregnancy characteristics**		
Gestational age at admission, mean ± SD (range)	32.9 ± 6.1 (24–40)	30.7 ± 9 (12–38)
Gestational hypertension, *n* (%)	0	2/32 (6.25%)
Preeclampsia, *n* (%)	0	4/32 (12.5%)
Retrochorionic hematoma, *n* (%)	0	1/32 (3.13%)
**Delivery characteristics**		
Gestational age at the delivery, mean ± SD (range)	37.4 ± 2.4 (30–40)	37.4 ± 2.1 (29–41)
Cesarean section, *n* (%)	1/12 (8.3%)	7/32 (28.7%)
Vacuum extraction delivery, *n* (%)	1/12 (8.3%)	2/32 (6.25%)

**Table 2:** Characteristics of the hemostasis system of pregnant women.

Parameter	Control (*n* = 12)	Complicated (*n* = 32)
Fibrinogen, mg/mL	3.7 ± 0.7 (2.6–4.8) *	4.7 ± 0.9 (3.2–6.7) *
PT INR	0.93 ± 0.10 (0.77–1.00)	1.09 ± 0.11 (0.78–1.43)
APTT, sec	31 ± 4 (26–35)	30 ± 4 (25–40)
Protein C, %	86.3 ± 16.0 (68.0–110.0)	86.6 ± 13.0 (65.0–120.0)
Platelet count, ×10^9^/L	217 ± 83 (125–348)	227 ± 55 (154–400)
Rate of ADP-induced platelet aggregation, %	37 ± 9 (23–52) *	47 ± 14 (20–80) *
Lag-phase of collagen-induced platelet aggregation, sec	192 ± 74 (65–300)	163 ± 87 (40–360)

Data is presented as mean ± SD (min-max range). Statistical significance of differences was analyzed by using the Mann–Whitney test. * *p* ≤ 0.05.

## Data Availability

The data supporting the findings of this study are available from the authors upon request, subject to approval by the Ethics Commission of the Shupyk National Medical Academy of Postgraduate Education. Due to ethical restrictions, the data are not publicly available.

## Abbreviations

The following abbreviations are used in this manuscript:

PAI	Plasminogen activator inhibitor
PA	Plasminogen activator
tPA	Tissue-type plasminogen activator
uPA	Urokinase-type plasminogen activator
aPTT	Activated partial thromboplastin time
PT	Prothrombin time
ROTEM	Rotational thromboelastography
ELISA	Enzyme-linked immunoassay
TBS	Tris-buffered saline
ECL	Enhanced chemiluminescence
